# Gridded Population Maps Informed by Different Built Settlement Products

**DOI:** 10.3390/data3030033

**Published:** 2018-09-04

**Authors:** Fennis J. Reed, Andrea E. Gaughan, Forrest R. Stevens, Greg Yetman, Alessandro Sorichetta, Andrew J. Tatem

**Affiliations:** 1 Geography and Geosciences, University of Louisville, Louisville, KY 40292, USA; p0reed02@louisville.edu; 2 CIESIN, Columbia University, Palisades, NY 10964, USA; 3 WorldPop, Department Geography and Environment, University of Southampton, Southampton SO17 1B, UK; 4 Flowminder Foundation, SE-11355 Stockholm, Sweden

**Keywords:** gridded population distribution, geography, built areas, remote sensing, geographic information systems, random forest, regression, binary dasymetric

## Abstract

The spatial distribution of humans on the earth is critical knowledge that informs many disciplines and is available in a spatially explicit manner through gridded population techniques. While many approaches exist to produce specialized gridded population maps, little has been done to explore how remotely sensed, built-area datasets might be used to dasymetrically constrain these estimates. This study presents the effectiveness of three different high-resolution built area datasets for producing gridded population estimates through the dasymetric disaggregation of census counts in Haiti, Malawi, Madagascar, Nepal, Rwanda, and Thailand. Modeling techniques include a binary dasymetric redistribution, a random forest with a dasymetric component, and a hybrid of the previous two. The relative merits of these approaches and the data are discussed with regards to studying human populations and related spatially explicit phenomena. Results showed that the accuracy of random forest and hybrid models was comparable in five of six countries.

## 1. Summary

As of 2017, the global human population is estimated to be near 7.6 billion, demonstrating a global population growth of roughly 200 million since 2015 [1]. By 2050, the human population is estimated to increase by at least 2 billion, with the largest global population growth per continent in Africa and Asia [1]. This change is implicitly associated with increasing rates of urbanization, which are seen most prominently in highly populated low- and middle-income countries, which together account for 37% of projected population growth into 2050 [2]. These global patterns of population change highlight the need for spatially explicit and comparable high-resolution gridded population datasets that accurately depict the spatial distribution of the residential human population and inform many fields, including infectious disease assessment [3–5], disaster response [6], adaptive strategies towards climate change mitigation [7,8] and many of the Millennium Development Goals [9]. This need is met by a broad variety of gridded population techniques.

However, gridded population techniques vary greatly in their methods, ancillary inputs, complexity, and resolution of interest [10]. Generally, gridded population techniques can be categorized into top-down and bottom-up approaches, wherein bottom-up approaches refer to calculating population size from ancillary data, whereas top-down estimates start with census data and try to disaggregate population further within units. Among the most straightforward top-down approaches are areal weighting, in which population is distributed uniformly across a continuous surface, as used in the Gridded Population of the World (GPW) v2-4 [11–13]. A modification of this technique called pycnophylactic interpolation proportionately distributes population along the edges of administrative units, as applied in GPW v1 [14]. A dasymetric mapping approach refines estimates by distributing population onto a weighted ancillary feature classification [15,16], as seen in the Global Rural Urban Mapping Project (GRUMP) and AfriPop and AsiaPop projects [4,5]. Dasymetric approaches have also been constrained in some cases to limit redistribution to certain areas and exclude it from others using a mask (i.e., binary features of land cover class, etc.) [16,17]. The most statistically advanced models of population redistribution are classified as smart interpolation [18], in which extensive ancillary inputs such as night-time lights, land cover, and topography provide a weighting scheme to redistribute population counts proportional to weights at grid-cell level [6,10,19]. In most cases, weighting layers are then used in dasymetric redistribution to constrain the total count within a known area, such as an administrative or census unit, to a population count for that areal unit [20]. While these methods are preferable for supporting disaster response and health applications, other non-modelled datasets such as GPW are still preferable for exploring the relationships between covariates [21]. Each method is used and demonstrates distinct strengths and weaknesses dependent on the objective of the study, the scale of the analysis, and data availability.

This paper presents the results of three different modeling approaches using three different high-resolution built-area datasets. Population was disaggregated using a representative selection of low- to middle-income countries, chosen for their high number of recent census administrative units, availability of ancillary inputs, and frequent exclusion from methods applied in higher income countries. The nine different gridded population datasets are available for six different countries for a total of 54 datasets at three arc second resolutions (~100 m at the equator).

## 2. Data Description

**Figure 4:**
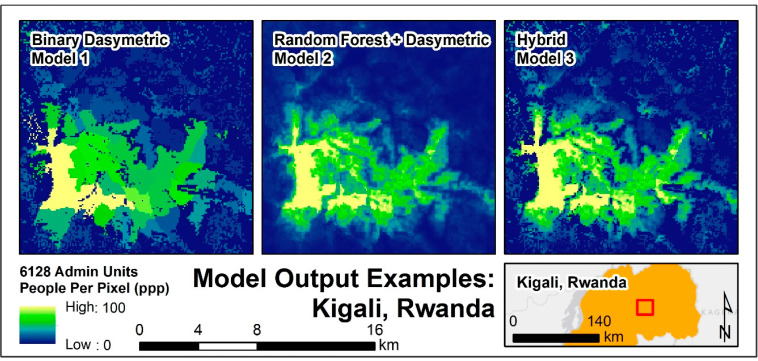
An example of the three primary model types and the rasters they produce for Kigali, Rwanda. Pictured built area extent on models 1 and 3 is the combination layer described in Section 3.1.2.

This dataset provides a set of 54 different high-resolution, gridded population raters produced for the purposes of methodological and built area data product comparison. Gridded products represent population as people per pixel (ppp) at ~100 m resolution for recent census years in select countries. This includes Madagascar, Rwanda, and Malawi from Africa, Nepal, and Thailand from Southeast Asia, and Haiti from the Caribbean. The gridded population datasets depict population distribution under the constraints of 3 different approaches explored in Table 3. Population estimates are presented in GeoTIFF format along with corresponding metadata, covariate importance, explanations of variance, and model accuracy assessment where appropriate. Examples of model outputs are previewed in Figure 4.

**Table 3:** Model enumeration and brief descriptions, indicating the number of resulting maps and built area restrictions. Ordered by increasing complexity.

Model	Name	Description	Raster Type	Output Maps
1	Binary Dasymetric	Redistribution of population into built areas.	Built Area Restricted	24
2	Random Forest + Dasymetric	Redistribution of population across weighted surface.	Continuous	6
3	Hybrid	Redistribution of population into weighted built areas.	Built Area Restricted	24

## 3. Methods

### 3.1. Preprocessing of Input Data

#### 3.1.1 Census Data

**Figure 1:**
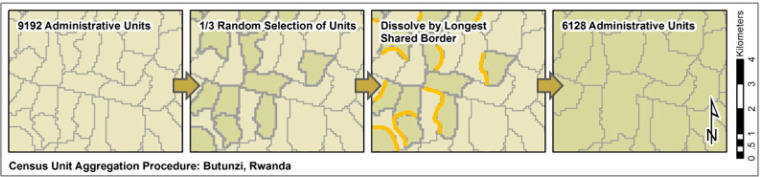
Census unit aggregation procedure in which 1/3 of the finest available units are randomly selected independent of spatial size or any other stratification and merged with its neighbor with the longest shared border until the target 2/3 census count is reached.

We use census data that represents the finest spatial resolution and most contemporary data that were publically available at the time of analysis. Retrieval of census data is made on request from country-specific National Statistics Offices. Census data are then matched to a country-specific GIS administrative level from GDAM (https://gadm.org/index.html) that is specific to the region and not comparable to units of the same level in different countries (Table 1) [22]. To ensure a level of comparability between countries, the Average Spatial Resolution (ASR) was calculated as the square root of its surface area divided by the number of administrative units, representing the effective resolution units within the country [4]. All models were run using a 2/3 aggregate of the finest available census data, in which a 1/3 random selection of units was dissolved with the neighbor sharing the longest border, as outlined in Figure 1.

**Table 1:** Census data for the six sampled countries and supporting data for finest available and aggregate products. Each model is built using the aggregate data, while finest available census units are reserved for accuracy assessment.

Type	Country	ISO	Census Year (Adm. Lvl.)	Admin Units	Total Pop	ASR
Finest Available	Haiti	HTI	2015 (3)	570	10,911,819	6.9
	Madagascar	MDG	2006 (4)	17,459	20,966,899	5.8
	Malawi	MWI	2008 (3)	12,666	13,053,968	2.7
	Nepal	NPL	2011 (4)	36,042	26,246,586	2.0
	Rwanda	RWA	2002 (4)	9192	9,482,511	1.7
	Thailand	THA	2010 (3)	7416	64,978,504	8.3
2/3 Aggregate	Haiti	HTI	2015	380	10,911,819	8.4
	Madagascar	MDG	2006	11,639	20,966,899	7.1
	Malawi	MWI	2008	8444	13,053,968	3.4
	Nepal	NPL	2011	24,028	26,246,586	2.5
	Rwanda	RWA	2002	6128	9,482,511	2.0
	Thailand	THA	2010	4944	64,978,504	10.2

#### 3.1.2 Built Area Data

For the purposes of this study, the term Built Area is used to describe both urban and built-up datasets, all of which are assumed to be indicative of human settlement. To test the effectiveness of combined dasymetric and random forest methods, we chose three built area datasets obtained using different remote sensing techniques with different spatial resolutions and criteria under which built-area is sensed. These publically available datasets include World Settlement Footprint (WSF), Global Human Settlement Layer (GHSL), and the Facebook Connectivity Lab’s High-Resolution Settlement Layer (HRSL) (Table 2).

**Table 2:** Three primary built/human settlement datasets and supporting information. GHSL and HRSL datasets are accessible from their respective portals, while WSF is available upon request [23].

Built Dataset	Year	Source	Nominal Resolution	Citation
WSF	2015	Landsat 8, Sentinel1	10 m	[24]
GHSL	2014	Landsat 8	38 m	[25]
HRSL	2015	DigitalGlobe	0.5 m	[26]

The first, World Settlement Footprint (WSF), represents a global coverage of earth’s land surface from the German Space Agency (DLR) Earth Observation Center based on Landsat 8 and Sentinel 1 optical and radar imagery for 2014–2015. The initial dataset was retrieved through personal communication with Thomas Esch and Mattia Marconcini and represents an initial version prior to public release [23,27]. Second, the Global Human Settlement Layer (GHSL) represents a global built-up dataset that focuses on three primary products: built-up areas, population grids, and urban/rural classification. The derived built area classifications use a combination of supervised and unsupervised procedures on the panchromatic channel of Landsat 8. Three land cover types are identified over four primary epochs, as informed by ancillary data from GHSL partners [25]. The global GHSL product is available on a global scale through the European Joint Research Center [28]. For the Facebook Connectivity Lab population product, distribution is determined using a combination of supervised classification and computer vision techniques on composited DigitalGlobe imagery [29]. Population distribution products may be downloaded for a limited number of countries as GeoTIFFs from CIESIN/FCL’s associated High Resolution Settlement Layer (HRSL) project [26]. It is worth noting that the proposed built datasets make no distinction between residential and commercial features, as limited by their remotely sensed methodology.

#### 3.1.3 Additional Ancillary Data

A wide range of ancillary data are used as explanatory variables of the random regression forest used in Models 2 and 3, as outlined in Table 3. While the most recent and detailed covariates will produce the best models [20], the best data is often regional and not consistently available across the study area. Thus, the ancillary data products used represent readily available, high-quality data that was present for all countries. Three types of covariate data include categorical rasters, continuous rasters, and converted vector data as outlined in Table 4.

**Table 4:** Covariates and data sources included in the random forest. Nominal resolutions noted with ‘as’ represent the unit arcseconds.

	**Description**	**Data Source, Year**	**Nominal Resolution**	**Citation**
**Categorical**	Cultivated Terrestrial Lands Woody/Trees Shrubs Herbaceous Other Terrestrial Vegetation Aquatic Vegetation Urban Area Bare Area Waterbodies	ESA CCI Land cover, 2010	10 arc-second	[30]
**Continuous Raster**	Lights at Night Mean Temperature Mean Precipitation Elevation Slope Built Distance to Outer Edge Built Distance to Outer Edge Built Distance to Outer Edge	Suomi VIIRS-Derived, 2012 WorldClim/BioClim, 1950–2000 WorldClim/BioClim, 1950–2000 HydroSHEDS, 2000 HydroSHEDS, 2000 WSF, 2015 GHSL, 2014 HRSL, 2015	15 arc-second 30 arc-second 30 arc-second 3 arc-second 10 m 38 m 5 m	[31] [32] [33] [24] [25] [26]
**Converted Vector**	Generic Populated Places Distance to Protected Areas Distance to Roads Distance to Rivers/Streams Distance to Waterbodies Cities Villages Buildings	VMAP0 merged, 1979–1999 WDPA, IUCN, 2012 OSM, 2017 OSM, 2017 OSM, 2017 OSM, 2017 OSM, 2017 OSM, 2017	NA	[34] [35] [36]

### 3.2 Data Production Workflow

**Figure 2:**
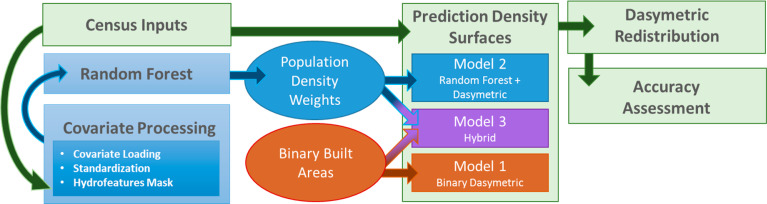
Workflow for generating the population distribution maps.

The following section outlines the open-access archive of comparable, high-resolution datasets of gridded population distribution for the countries of Haiti (HTI), Madagascar (MDG), Malawi (MWI), Rwanda (RWA), Nepal (NPL), and Thailand (THA). These countries represent criteria of comparable human distribution, heterogeneous land-cover types, and diverse continental representation. Figure 2 highlights the production of population estimates from the three models, broadly categorized into five stages.

The approach utilized here is adapted from published WorldPop random forest methodology that has been altered to suit this study needs [20]. For an in-depth analysis of programmatic operation, please refer to the procedural documents stored in [37]. The methods and scripts presented in this paper are from R 3.4.1, Python 2.7.8, and ESRI ArcMap 10.3.1.

The covariate selection and data preparation step has three primary phases of preparation, including built data processing, covariate standardization, and hydrofeature mask creation.

**Figure 3:**
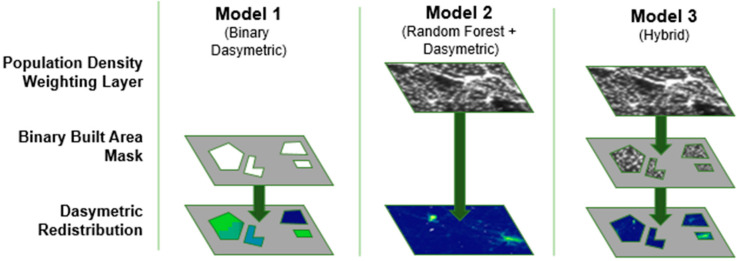
Model enumeration and visual representation of feature overlays used to produce output datasets by means of dasymetric redistribution. Ordered by increasing complexity.

First, we process the three built areas mentioned in Table 2 into binary built feature classifications. Resampling via presence/non-presence occurs on the binary masks to create a consistent ~100 m resolution and standardized projection (WGS 84 geographic coordinate system) prior to model application. It is worth noting that the described preparation here applies only for those built areas that will be used to constrain the binary dasymetric and hybrid models (Table 3, Figure 3), and that remaining covariates are manipulated in the parameterization of the random forest model, as described in Forrest et al. 2015 [20]. In addition to the independent built area layers, a fourth built area layer representing a combination of WSF, GHSL, and HRSL datasets provided a final dataset for comparison. By combining all built features, we increase the chance of false positives but simultaneously minimize errors of omission present in other built products.

Next, we cluster covariates into three groups depending on their subsequent transformations (Table 4). For example, the multi-class ESA land cover product classifications were separated into individual feature types and transformed using a distance to outer edge (DTE) calculation in ArcMap [30]. To produce the DTE covariate, the target feature is loaded at ~100 m resolution, refined to show the feature class in question if multiple classifications are present and re-projected to a region specific UTM. The same distance to outer edge calculation was also used in the preparation of the primary built areas as specified in Table 4. Final covariates products match in regional extent, spatial resolution (100 m resolution), and country-specific UTM projection.

The last component is generating a hydrofeatures mask based on the European Space Agency’s land use classification product [30] and processed as a binary raster with an 8 km buffer. By including sufficiently over-estimated borders, we ensure the combined extent of all stacked covariates will be identical and exclude additional features that might occur within the buffered boundary. The mask also acts to exclude a consistent representation of water features across the covariate stack. This is necessary, because while the study area is artificially bounded, the processes are not [38].

### 3.3 Model Types and Construction

We use three different models and the four built area configurations across six different countries to produce 54 models (Table 3). The first model type (Model 1, Figure 3) represents a simple binary dasymetric approach, in which census counts are disaggregated into pixels coincident with built areas defined by a given built product. To address the issue of census units with no built pixels, an iterative set of selections and redistributions mitigate the potential of under-estimating the population [37]. Figure 4, Model 1, demonstrates the visible boundary of built area constraint, in addition to the visible difference in population along administrative unit boundaries.

The second model (Model 2, Figure 3) creates a population density-weighting surface based on a random forest (RF) statistical model, which is explained further in Stevens et al. 2015 [20]. RFs are robust to noise, small sample sizes, and over-fitting, requiring minimal user parameterization [39, 40]. The three primary parameters include the number of covariates to be selected at each node, the number of trees in the forest, and the number of observations allowed in the terminal nodes of each decision tree [39]. Specifically, for the approach outlined in Reed et al. [41], we generated a forest of 500 individual trees, based on the results of multiple experimental runs to produce stable and minimized out-of-bag error predictions [37]. The RF model produces a population density estimation grid used to dasymetrically redistribute the population counts across the entire continuous weighting layer. Figure 4, Model 2 demonstrates no visible boundary of built area constraint and shows no stark boundaries between census administrative units.

Last, the third model (Model 3, Figure 3) uses the population density-weighting surface generated in Model 2 but restricts the redistribution of census data to built area grid cells. In doing so, areas excluded from the built classification are given a population count of 0, constraining where people can be located while maintaining the predictive detail of the random forest (Figure 2). Figure 4, Model 3 shows the same patterning in Model 2 but with the built area distributional constraints of Model 1.

### 3.4 Technical Validation

To assess the accuracy of each model, population based on a two-thirds aggregate of available administrative units at the finest level was resampled in Python 2.7.8 by dissolving boundaries with the longest shared border, sorted randomly without spatial consideration. These final mapping products are then compared to the finest level of census data available for a given country by summing gridded population estimates within each administrative unit [20]. The statistical measures include the root mean squared error (RMSE), percent root mean squared error (%RMSE), and the mean absolute error (MAE) [42].

### 3.5 Assessment of Gridded Population Datasets

Accuracy assessment of each map featured a suite of error metrics, including the RMSE and MAE for both population counts and density. Results show a consistent decrease in error relative to model complexity, with a few exceptions (Table 5). Those exceptions, as well as variation in accuracy for the more complex approaches, is ultimately dependent on the quality of the underlying RF model, which is a function of the nominal resolution captured by input census data and covariates.

**Table 5:** Error metrics for each of the 52 maps. Tables are shaded to indicate increasing methodological complexity. Values highlighted in red represent minimum error. Labeled as follows a: Haiti, b: Madagascar, c: Malawi, d: Nepal, e: Rwanda, f: Thailand.

	Model	Built Area	RMSE	MAE	RMSE Density	MAE Density			Model	Built Area	RMSE	MAE	RMSE Density	MAE Density	
(a)	Dasymetric Masked Dasymetric Masked Dasymetric Masked Dasymetric Masked	HRSL GHSL WSF COMBO	12861.2 13733.9 12206.1 13148.8	3281 4807.7 4051.2 3341.2	8.1 8.5 8.3 8.3	1.6 2.1 1.8 1.6	**Haiti** **Haiti**Haiti	(b)	Dasymetric Masked Dasymetric Masked Dasymetric Masked Dasymetric Masked	HRSL GHSL WSF COMBO	777.4 1142.1 887.4 835.1	245.9 401 371.6 252.9	32.9 33.5 34.3 36.1	3.9 4.8 4.3 4.3	**Madagascar** **Madagascar**Madagascar
	Random Forest + Dasymetric		11083.9	3021.8	7.3	1.5			Random Forest + Dasymetric		934.5	287.9	37.6	4.7	
	Hybrid	HRSL	11935.6	3061.9	7.9	1.5			Hybrid	HRSL	727.2	256.6	37.1	3.9	
	Hybrid	GHSL	12823.1	4779	8.1	2			Hybrid	GHSL	1130.1	403.3	33.1	4.8	
	Hybrid	WSF	12267.5	4548.4	8.1	2			Hybrid	WSF	897.2	380.4	33.7	4.3	
	Hybrid	COMBO	11897.6	3116.8	7.9	1.5			Hybrid	COMBO	782.4	271.4	39.3	4.2	
(c)	Dasymetric Masked Dasymetric Masked Dasymetric Masked Dasymetric Masked	HRSL GHSL WSF COMBO	549.1 722.5 700.5 615.4	225.2 337.9 345 238.3	31.1 28 27.5 30.4	5 5.5 5.4 5.3	**Malawi** **Malawi**Malawi	(d)	Dasymetric Masked Dasymetric Masked Dasymetric Masked Dasymetric Masked	HRSL GHSL WSF COMBO	456.3 638.2 533 452	176.2 205 217.8 173.6	22 27.4 23.6 21.9	3.7 4.6 4.4 3.7	**Nepal** **Nepal**Nepal
	Random Forest + Dasymetric		567.6	213.6	27.7	4.8			Random Forest + Dasymetric		412.5	140.8	21.8	3.4	
	Hybrid	HRSL	529	233.7	30.2	4.9			Hybrid	HRSL	452.6	186.7	22.4	3.9	
	Hybrid	GHSL	699.1	340.5	27.1	5.5			Hybrid	GHSL	645.5	209	27.6	4.6	
	Hybrid	WSF	705.9	354.3	27.1	5.5			Hybrid	WSF	540.1	224.5	23.9	4.6	
	Hybrid	COMBO	545.3	236.2	28.5	4.9			Hybrid	COMBO	448.5	185.2	21.9	3.8	
(e)	Dasymetric Masked Dasymetric Masked Dasymetric Masked Dasymetric Masked	HRSL GHSL WSF COMBO	390.9 593.3 575.1 398.9	146.7 286.3 271.7 149.1	11.3 11.7 11.9 11.5	1.7 2.7 2.7 1.7	**Rwanda** **Rwanda**Rwanda	(f)	Dasymetric Masked Dasymetric Masked Dasymetric Masked Dasymetric Masked	HRSL GHSL WSF COMBO	4040.9 4048.7 3986.7 4257.1	1160.3 1493.2 1208.1 1183.5	9.8 9 9.4 10.9	1.5 1.5 1.5 1.6	**Thailand** **Thailand**Thailand
	Random Forest + Dasymetric		343.4	110.3	11.1	1.4			Random Forest + Dasymetric		3802.9	1139.5	9.9	1.4	
	Hybrid	HRSL	376.3	153.2	10.7	1.7			Hybrid	HRSL	3697.2	1278.9	8.6	1.3	
	Hybrid	GHSL	595.7	291.4	11.4	2.7			Hybrid	GHSL	4279	1789	8.3	1.6	
	Hybrid	WSF	579	273.9	11.6	2.7			Hybrid	WSF	3932.4	1462.8	8.3	1.4	
	Hybrid	COMBO	386.1	157.7	11	1.7			Hybrid	COMBO	3809.1	1299.5	9.6	1.4	
	Model	Built Area	RMSE	MAE	RMSE Density	MAE Density			Model	Built Area	RMSE	MAE	RMSE Density	MAE Density	

**Table 6:** Variance explained captured in the random forest models of each sampled country.

Country	Variance Explained	Country	Variance Explained
Haiti	52.4	Nepal	82.12
Madagascar	78.96	Thailand	84.49
Malawi	72.27	Rwanda	73.07

**Figure 5:**
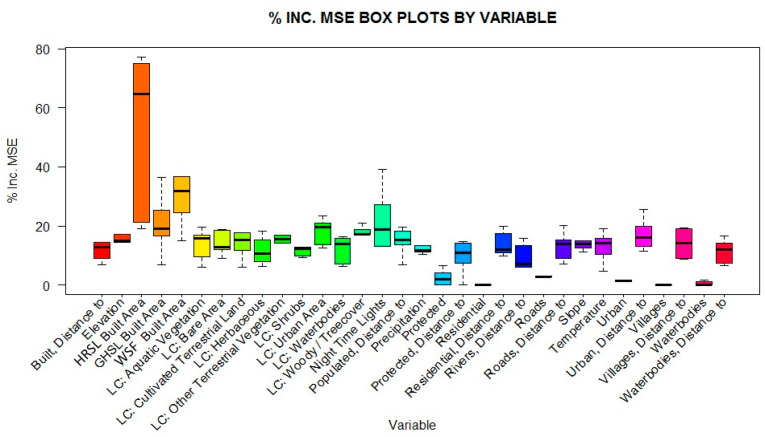
Box plots of global variable importance presented as mean squared error for each covariate class. The median is represented by the black bar, while the whiskers represent the min/max values within 1.5× inter-quartile range. Variables sourced in Table 4.

The random forest model that produces the population density-weighting layer for the RF and Hybrid approaches has a variance explained for each country noted in Table 6. The variance explained fell consistently between 72.3% and 84.5%. The only exception was Haiti, where only 52.4% of variance could be explained due to an already low number of large census administrative units, which is known to decrease the predictive capacity of the models (Table 6) [12,20,43]. In terms of covariate importance, the HRSL built area delineations had the greatest covariate importance across all countries (Figure 5).

## 4. User Notes

The datasets presented in this paper facilitate comparisons and considerations of different approaches to the production of gridded population data. When producing such data, it is worth assessing the underlying built data and associated population densities to assess whether a binary dasymetric or hybrid approach may be more appropriate than statistical or smart interpolation models. The datasets presented here are endogenous and should not be used to explore relationships and correlations between the ancillary datasets and the resulting population distribution [4]. Please see Reed et al. for a full analysis of environmental queues for population model selection [41]. The provided dataset is limited by the ~100 m spatial resolution, which does not represent the same pattern at alternate scales. Additionally, all built areas were resampled from their finest available product by presence/non-presence and are not representative of spatial grain at the time of sensing. Finally, model results are limited by the quality of inputs and are expected to perform more accurately if parameterized with the finest available census data and regionally specified covariates. Processing times for each model were dependent on computing architecture, the area of the country covered that determines the memory demands for processing the rasters, and the total number of areal units processed during zonal statistics calculations. The processing time, however, is also highly dependent on the number of parallel processing units available. Both the model estimation for Random Forests and the per-pixel predictions can be highly parallelized, allowing for total processing times to scale directly with computing resources.

## Supplementary Materials

The full body of WorldPop processing is associated with the Stevens et al. publication [20] and a more in-depth analysis of these specific products is outlined in Reed et al., in review. Scripts written for dasymetric models and documentation can be found at the corresponding DOI, which may be explored by selecting ‘Browse Individual Files’ at the base of the page [37].
