# Supplementary material for: Gridded Population Maps Informed by Different Built Settlement Products
Source: Data (Basel). 2018 Sep 4;3:33. doi: 10.3390/data3030033 (PMC7680951; doi:10.3390/data3030033)
Supplement: Supplementary file 1 [file DATA-03-03-033-s001.pdf]

Effectiveness of remotely Sensed Built Areas for Constraining and Modelling Gridded Population Estimates - Script Samples and data

These data present the effectiveness of three different high-resolution built area datasets for producing gridded population estimates through the dasymetric disaggregation of census counts in Haiti, Malawi, Madagascar, Nepal, Rwanda, and Thailand. Modeling techniques include a binary dasymetric redistribution, random forest with dasymetric component, and a hybrid of the previous two.

Region : Africa

DOI : 10.5258/SOTON/WP00643

Date of production : 2018-11-01

Recommended citation

CITATION: WorldPop. 2018. Effectiveness of Remotely Sensed Built Areas for Constraining and Modelling Gridded Population Estimates - Script Samples and Data. University of Southampton.

Pdf file : Get pdf file

Data Files :

Download Entire Dataset / **1.94 GB** Browse Individual Files

Download individual files below

×

Haiti.7z	45.13 MB
Madagascar.7z	779.83 MB
Nepal.7z	228.52 MB
Thailand.7z	747.22 MB
Rwanda.7z	44.41 MB
Source_code.7z	0.78 MB
Malawi.7z	144.60 MB

Close

WorldPop datasets are available under the Creative Commons Attribution 4.0 International License. This means that you are free to share (copy and redistribute the material in any medium or format) and adapt (remix, transform, and build upon the material) for any purpose, even commercially, provided attribution is included (appropriate credit and a link to the licence).

×
